# Docosahexaenoic Acid Inhibits Inflammation-Induced Osteoclast Formation and Bone Resorption *in vivo* Through GPR120 by Inhibiting TNF-α Production in Macrophages and Directly Inhibiting Osteoclast Formation

**DOI:** 10.3389/fendo.2019.00157

**Published:** 2019-03-15

**Authors:** Akiko Kishikawa, Hideki Kitaura, Keisuke Kimura, Saika Ogawa, Jiawei Qi, Wei-Ren Shen, Fumitoshi Ohori, Takahiro Noguchi, Aseel Marahleh, Yasuhiko Nara, Atsuhiko Ichimura, Itaru Mizoguchi

**Affiliations:** ^1^Division of Orthodontics and Dentofacial Orthopedics, Department of Translational Medicine, Tohoku University Graduate School of Dentistry, Sendai, Japan; ^2^Department of Biological Chemistry Graduate School of Pharmaceutical Sciences, Kyoto University, Kyoto, Japan; ^3^Keihanshin Consortium for Fostering the Next Generation of Global Leaders in Research, Kyoto, Japan

**Keywords:** osteoclast, DHA, LPS, mouse, GPR120

## Abstract

Docosahexaenoic acid (DHA) is an n-3 fatty acid that is an important structural component of the cell membrane. DHA exerts potent anti-inflammatory effects through G protein-coupled receptor 120 (GPR120), which is a functional receptor for n-3 fatty acids. DHA also regulates osteoclast formation and function. However, no studies have investigated the effect of DHA on inflammation-induced osteoclast formation *in vivo*. In the present study, we investigated whether DHA influences osteoclast formation, bone resorption and the expression of osteoclast-associated cytokines during lipopolysaccharide (LPS)-induced inflammation *in vivo*, and then we elucidated the underlying mechanisms by using *in vitro* experiments. *In vitro* experiments revealed both receptor activator of NF-kB ligand (RANKL)- and tumor necrosis factor-α (TNF-α)-induced osteoclast formation was inhibited by DHA. Supracalvarial administration of LPS with or without DHA was carried out for 5 days and then the number of osteoclasts, ratio of bone resorption pits and the level of type I collagen C-terminal cross-linked telopeptide were measured. All measurements were significantly lower in LPS+DHA-co-administered mice than LPS-administered mice. However, this DHA-induced inhibition was not observed in LPS-, DHA-, and selective GPR120 antagonist AH7614-co-administered mice. Furthermore, the expression of RANKL and TNF-α mRNAs was lower in the LPS+DHA-co-administered group than in the LPS-administered group *in vivo*. TNF-α mRNA levels were decreased in macrophages co-treated with LPS+DHA compared with cells treated with LPS *in vitro*. In contrast, RANKL mRNA expression levels from osteoblasts co-treated with DHA and LPS *in vitro* were equal to that in cells treated with LPS alone. Finally, the inhibitory effects of DHA on osteoclast formation *in vitro* were not observed by using osteoclast precursors from GPR120-deficient mice, and inhibition of LPS-induced osteoclast formation and bone resorption by DHA *in vivo* was not observed in GPR120-deficient mice. These results suggest that DHA inhibits LPS-induced osteoclast formation and bone resorption *in vivo* via GPR120 by inhibiting LPS-induced TNF-α production in macrophages along with direct inhibition of osteoclast formation.

## Introduction

Osteoclasts, which are specialized bone resorbing cells derived from hematopoietic stem cells, play important roles in bone remodeling and in bone destruction in diseases such as rheumatoid arthritis, osteoporosis, and periodontal disease (1). Two important cytokines, receptor activator of NF-kB ligand (RANKL) and macrophage colony stimulating factor (M-CSF), are required for osteoclast formation and osteoclast function (2). M-CSF is also essential for proliferation of osteoclast precursors. Tumor necrosis factor-α (TNF-α) has also been reported to induce osteoclast formation both *in vitro* (3–5) and *in vivo* (6, 7). TNF-α is central to pathological bone disorders including inflammation (8).

Lipopolysaccharide (LPS), which is a major constituent of the cell wall of Gram-negative bacteria, induces inflammation and pathological bone destruction (9, 10). LPS also induces the production of pro-inflammatory cytokines, such as TNF-α from macrophages and other cells at sites of inflammation (11, 12). Furthermore, LPS stimulates osteoblasts to produce and express osteoclast-related cytokine RANKL (13). These cytokines have been linked to LPS-induced osteoclast formation and bone resorption in both *in vivo* and *in vitro* studies (9, 14).

It has been reported that polyunsaturated fatty acids confer some beneficial effects on cardiovascular diseases (15), autoimmune disorders and inflammatory disorders such as rheumatoid arthritis, inflammatory bowel disease, and dysmenorrhea (16, 17) and diabetes (18). Docosahexaenoic acid (DHA), a well-known dietary n-3 polyunsaturated fatty acid, is a long-chain polyunsaturated fatty acid that has 22 carbon atoms and 6 double bonds. DHA is used as a food supplement and has favorable effects against certain cancers, diabetes and cardiovascular diseases (19, 20).

G protein-coupled receptors (GPRs) play a pivotal role as signaling molecules for many cellular functions. G protein-coupled receptors are seven transmembrane domain receptors that regulate many physiological and pathological responses (21–24). It has been reported that free fatty acids can activate receptors GPR40 (free fatty acid receptor: FFAR1), GPR41 (FFAR3), GPR43 (FFAR2), GPR84, and GPR120 (FFAR4) and long-chain fatty acids can activate GPR40 and GPR120 (25, 26). GPR120, also known as free fatty acid receptor 4 (FFAR4), has also been implicated in homeostatic metabolic regulation in immune processes and inflammatory *in vivo* (27). Therefore, GPRs are leading targets for drug development for many human diseases. Especially, GPR120 has gathered attention owing to its potential role in the regulation of many inflammation-related diseases such as diabetes and obesity (21, 26–31).

It has been reported that dietary n-3 fatty acids inhibit bone loss in ovariectomized mice owing to their inhibitory effects on osteoclast formation (32). Some studies showed the inhibitory effects of DHA on osteoclast formation and activity *in vitro*. It has been reported that DHA inhibits osteoclast formation and function in human CD14+ monocytes (33). It has also been shown the differential effects of DHA on osteoclast formation in the murine monocytic cell line RAW 264.7 (34). These *in vitro* studies give some insight into the effects of DHA on osteoclast formation, however, the effects of DHA on osteoclast formation *in vivo* remain unclear. Furthermore, the effects of DHA through GPR120 on osteoclast formation *in vivo* have not been investigated.

In the present study, we showed the effects of DHA on LPS-induced osteoclast formation and bone resorption via GPR120 in a murine experimental model and elucidated the underlying mechanisms by using *in vitro* experiments.

## Materials and Methods

### Animals and Reagents

Eight- to ten-week-old male C57BL6/J mice were obtained from CLEA Japan (Tokyo, Japan). C57BL6 mice bearing the *Ffar4*-floxed gene were generated by an outsourced research company (Transgenic Inc., Fukuoka, Japan) as schematically illustrated in Supplementary Figure 1. In the conditional *Ffar4*-targeting vector designed according to the recombineering-based construct, two loxP sites were inserted into the introns immediately upstream and downstream of the exon 1 such that an FRT-containing neomycin resistance cassette was included in the loxP-flanked region. The targeting vector was introduced into ES cells, and homologous recombination-positive clones were isolated and used for generating germline-chimeric mice by the embryo aggregation method. Heterozygous mutant mice bearing the homologous mutation were produced and bred with the Flp mice (Jackson Laboratory, Bar Harbor, USA) to remove the neomycin resistance cassette. To generate systemic *Ffar*4 knock out mice [*Ffar*4^(dE1/dE1)^ mice], the floxed mice were crossed with transgenic mice expressing Cre -recombinase under the control of the chicken actin promoter (CAG). All animal care and experiments were conducted according to Tohoku University rules and regulations. Four mice were randomly distributed in each experimental group. DHA (Sigma-Aldrich, St Louis, MO, USA), *Escherichia coli* LPS (Sigma-Aldrich) and GPR120 antagonist AH7614 (Tocris Bioscience, Bristol, United Kingdom) were used in this study. For *in vitro* study, recombinant mouse RANKL (35) and TNF-α (6) were obtained as previously described, and recombinant mouse M-CSF was prepared from an M-CSF-expressing cell line (CMG14-12) (36).

### Histological Analysis

In a previous study, daily subcutaneous supracalvarial administration of 100 μg LPS to mouse calvariae for 5 days significantly induced osteoclast formation (37, 38). Therefore, we followed the same protocol, dose and LPS administration period in this study. Four mice were randomly distributed in each group and received daily subcutaneous injections of phosphate-buffered saline (PBS; negative control group), LPS (100 μg/day, positive control group), LPS (100 μg/day), and DHA (100 μg/day) with or without AH7614 (100 μg/day) and DHA (100 μg/day). *Ffar4*^(dE1/dE1)^ mice were subjected to daily subcutaneous injections of LPS (100 μg/day, positive control group) and LPS (100 μg/day) and DHA (100 μg/day). Calvariae were resected immediately after sacrifice on the 6th day. After fixation in 4% PBS-buffered formaldehyde, all calvariae were demineralized in 14% ethylenediaminetetraacetic acid (EDTA) for 3 days. After dehydration, calvariae were embedded in paraffin and sectioned perpendicular to the sagittal suture (5-μm thickness) using a microtome (Leica, Wetzlar, Germany). Sections were stained for tartrate-resistant acid phosphatase (TRAP) and hematoxylin counterstain as previously described (37, 38). Osteoclasts were identified as TRAP-positive cells containing three or more nuclei. The number of osteoclasts located at the mesenchyme of the sagittal suture was counted in all slides as described previously (37, 38). In addition, the percentage of interface of bone marrow space covered by osteoclasts and the number of osteoclast of interface of bone marrow space were histomorphometrically determined in specimens derived from each sample.

### Preparation of Osteoclast Precursors for Osteoclast Formation

Osteoclast formation was evaluated as previously described (37, 38). Briefly, femoral and tibial epiphyses from 8 to 10-week-old male C57BL/6J mice were cut, and then the bone marrow was flushed using a 25-gauge needle and syringe prefilled with alpha-modified minimal essential medium (α-MEM; Sigma-Aldrich). Harvested cells were then cultured in α-MEM containing 10% fetal bovine serum, 100 IU/mL penicillin G (Meiji Seika, Tokyo, Japan) and 100 μg/mL streptomycin (Meiji Seika) with M-CSF. Adherent cells were harvested using trypsin-EDTA solution (Sigma-Aldrich). Harvested cells were then cultured in the presence of M-CSF until they reached confluency. Cells were recognized as osteoclast precursors. Osteoclast precursors were seeded at 5 × 10^4^ cells per 200 μL of culture medium in a 96-well plate and cultured for 5 days in medium containing M-CSF (100 ng/mL); M-CSF (100 ng/mL) and RANKL (100 ng/mL) or TNF-α (100 ng/mL); M-CSF (100 ng/mL), RANKL (100 ng/mL), or TNF-α (100 ng/mL), and DHA (100 ng/mL) with or without AH7614 (100 ng/mL); or M-CSF (100 ng/mL) and DHA (100 ng/mL). After fixation with 4% PBS-buffered formaldehyde, cells were permeabilized with 0.2% Triton X-100 and TRAP staining was performed to visualize active osteoclasts. TRAP-positive cells containing three or more nuclei were counted under a light microscope (37, 38).

### Isolation of Murine Macrophages

Eight- to ten-week old C57BL/6J mice were sacrificed to obtain peritoneal macrophages; 5 mL sterile ice-cold PBS (pH 7.4) was injected into the peritoneal cavity and then the fluid was aspirated out to collect cells. Collected cells were incubated for 1 h and then non-adherent cells were removed. Adherent cells were cultured an additional 24 h and used as peritoneal macrophages (37, 38).

### RNA Preparation and Real-Time RT-PCR Analysis

Calvariae from *in vivo* experiments were frozen in liquid nitrogen and crushed by Micro Smash MS-100R (Tomy Seiko, Tokyo, Japan) in 800 μL TRIzol reagent (Invitrogen, Carlsbad, CA) for each sample. Total RNA was extracted with an RNeasy mini kit (Qiagen, Valencia, CA) according to the manufacturer's protocol. Osteoblasts (Cosmo Bio, Tokyo, Japan) or macrophages were cultured in culture medium supplemented with PBS; LPS (100 ng/mL); LPS (100 ng/mL); and DHA (100 ng/mL) with or without AH7614 (100 ng/mL); or DHA (100 ng/mL). After 24 h culture, total RNA was extracted from osteoblasts or peritoneal macrophages. cDNA was synthesized for each sample from 2 μg total RNA with oligo-dT primers (Invitrogen) and reverse transcriptase in a total volume reaction of 20 μL. To assess the gene expression value, the Thermal Cycler Dice Real Time System (Takara, Shiga, Japan) was used for real-time RT-PCR. Each reaction comprised a total volume of 25 μL containing 2 μL cDNA and 23 μL of a mixture of SYBR Premix Ex Taq (Takara) and 50 pmol/μL primers. PCR cycling conditions were as follows: 95°C for 10 s for initial denaturation followed by 45–60 amplification cycles, with each cycle comprising a denaturation step of 95°C for 5 s and then an annealing step of 60°C for 30 s. Relative expression levels of RANKL and TNF-α mRNAs were calculated by normalization to glyceraldehyde 3-phosphate dehydrogenase (GAPDH) mRNA levels. Primer sequences used for cDNA amplification were as follows: 5′-GGTGGAGCCAAAAGGGTCA-3′ and 5′-GGGG GCTAAGCAGTTGGT-3′ for GAPDH; 5′-AGGCGGTGC TTGTTCCTCA-3′ and 5′-AGGCGAGAAGATGATCTGA CTGCC-3′ for TNF-α; and 5′-CCTGAGGCCAGCCATTT- 3′ and 5′-CTTGGCCCAGCCTCGAT-3′ for RANKL as already reported (37, 38).

### Analysis of Bone Resorption

To evaluate the bone resorption area, calvariae were fixed in 4% PBS-buffered formaldehyde and then scanned with microfocus computed tomography (CT) (ScanXmate-E090; Comscan, Kanagawa, Japan). Three-dimensional images of the calvariae were prepared using TRI/3D-BON64 software (RATOC System Engineering, Tokyo, Japan), and the ratio of the bone resorption area to the total area was measured using ImageJ (NIH, Bethesda, MD) (37, 38).

### Measurement of Serum C-Terminal Telopeptide (CTX) of Type I Collagen

Serum was obtained from mice after 5 days of daily supracalvarial administration of PBS, LPS, LPS+DHA with or without AH7614 or DHA alone. Serum CTX levels were evaluated using a mouse CTX assay kit (IDS, Tyne and Wear, UK) by measuring absorbance at 450 nm using a microplate reader (Remote Sunrise; Tecan, Kawasaki, Japan) with a reference wavelength of 620 nm (37, 38).

### Statistical Analyses

All data are expressed as means ± standard deviation (SD). Differences between groups were assessed using Scheffe's test, one-way ANOVA and the Turkey-Kramer test. Statistical significance was assumed at a threshold of *p* < 0.05.

## Results

### DHA Inhibits RANKL- and TNF-α-Induced Osteoclast Formation via GPR120 Activation *in vitro*

**Figure 1:**
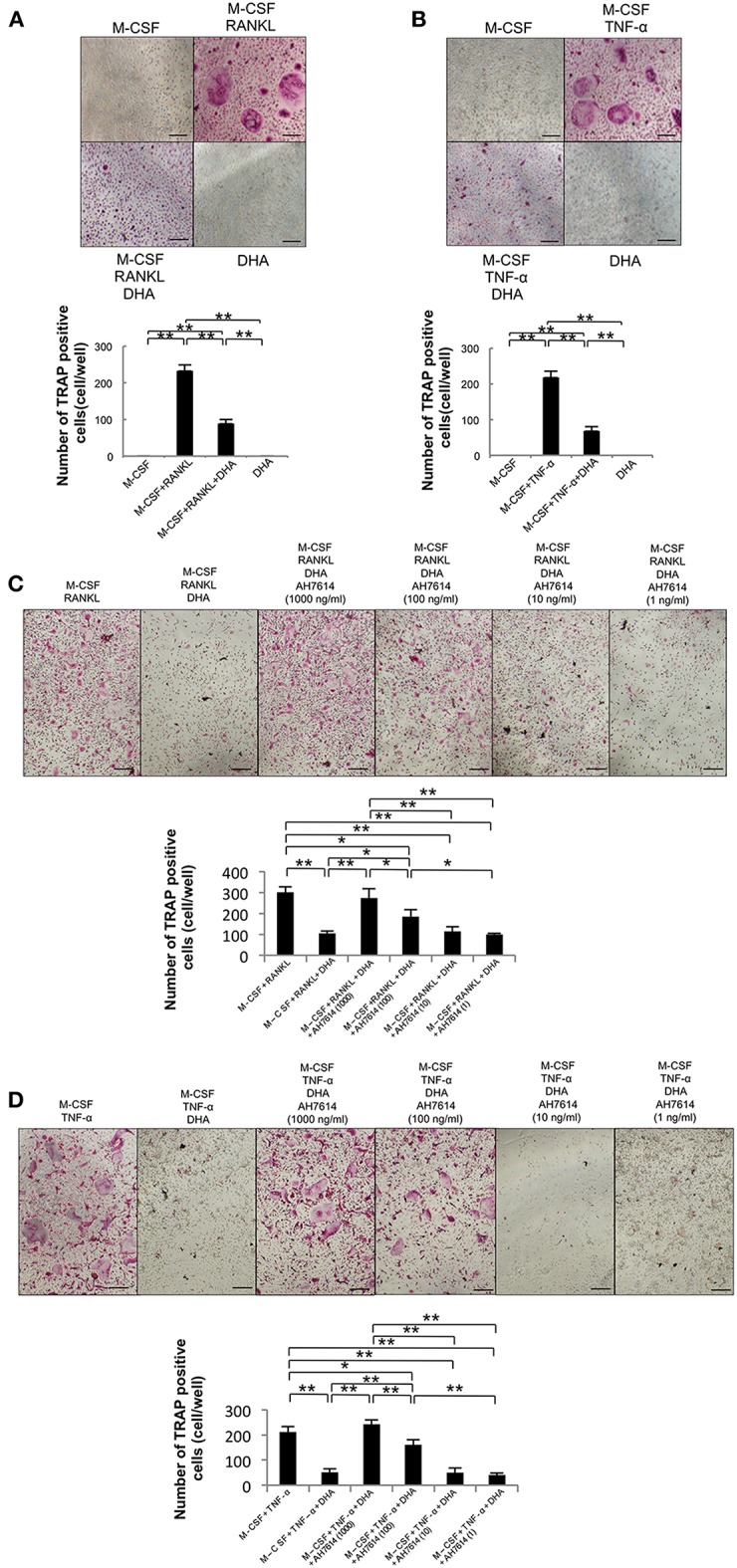
DHA inhibits RANKL- and TNF-α-induced osteoclast formation via GPR120 *in vitro*. **(A)** Microscopic images and numbers of TRAP-positive cells following RANKL-induced osteoclast formation. Osteoclast precursors were cultured with M-CSF, M-CSF+RANKL, M-CSF+RANKL+DHA, or M-CSF+DHA for 5 days, followed by staining with TRAP solution. **(B)** Microscopic images and numbers of TRAP-positive cells following TNF-α-induced osteoclast formation. Osteoclast precursors were treated with M-CSF, M-CSF+TNF-α, M-CSF+TNF-α+DHA, or M-CSF+DHA for 5 days, followed by staining with TRAP solution. **(C)** Microscopic images and numbers of TRAP-positive cells following RANKL-induced osteoclast formation under AH7614 treatment. Osteoclast precursors were cultured with M-CSF+RANKL, M-CSF+RANKL+DHA, or M-CSF+RANKL+DHA and various concentration of AH7614 (1,000, 100, 10, and 1 ng/mL). **(D)** Microscopic photos and numbers of TRAP-positive cells following TNF-α-induced osteoclast formation under AH7614 treatment. Osteoclast precursors were cultured with M-CSF+TNF-α, M-CSF+TNF-α+DHA, or M-CSF+TNF-α+DHA and various concentration of AH7614 (1,000, 100, 10, and 1 ng/mL). Data are expressed as means ± SD (*n* = 4; ^**^p < 0.01, ^*^p < 0.05). Statistical significance was determined by Scheffe's test. Scale bars = 100 μm.

First, we examined the influence of DHA on RANKL- and TNF-α-induced osteoclast formation to clarify whether DHA directly influences osteoclast precursor cells (Figures 1A,B). A large number of TRAP-positive cells were noted among osteoclast precursor cells cultured with M-CSF and RANKL or TNF-α. In contrast, the number of TRAP-positive cells was markedly decreased when cells were cultured with M-CSF and RANKL or TNF-α with DHA. Then, we investigated whether the inhibitory effect of DHA is diminished by GPR120 antagonist AH7614 (Figures 1C,D). AH7614 dose-dependently increased the number of TRAP-positive cells compared with cells cultured with M-CSF and RANKL or TNF-α with DHA.

### DHA Inhibits LPS-Induced Osteoclast Formation via GPR120 Activation *in vivo*

**Figure 2:**
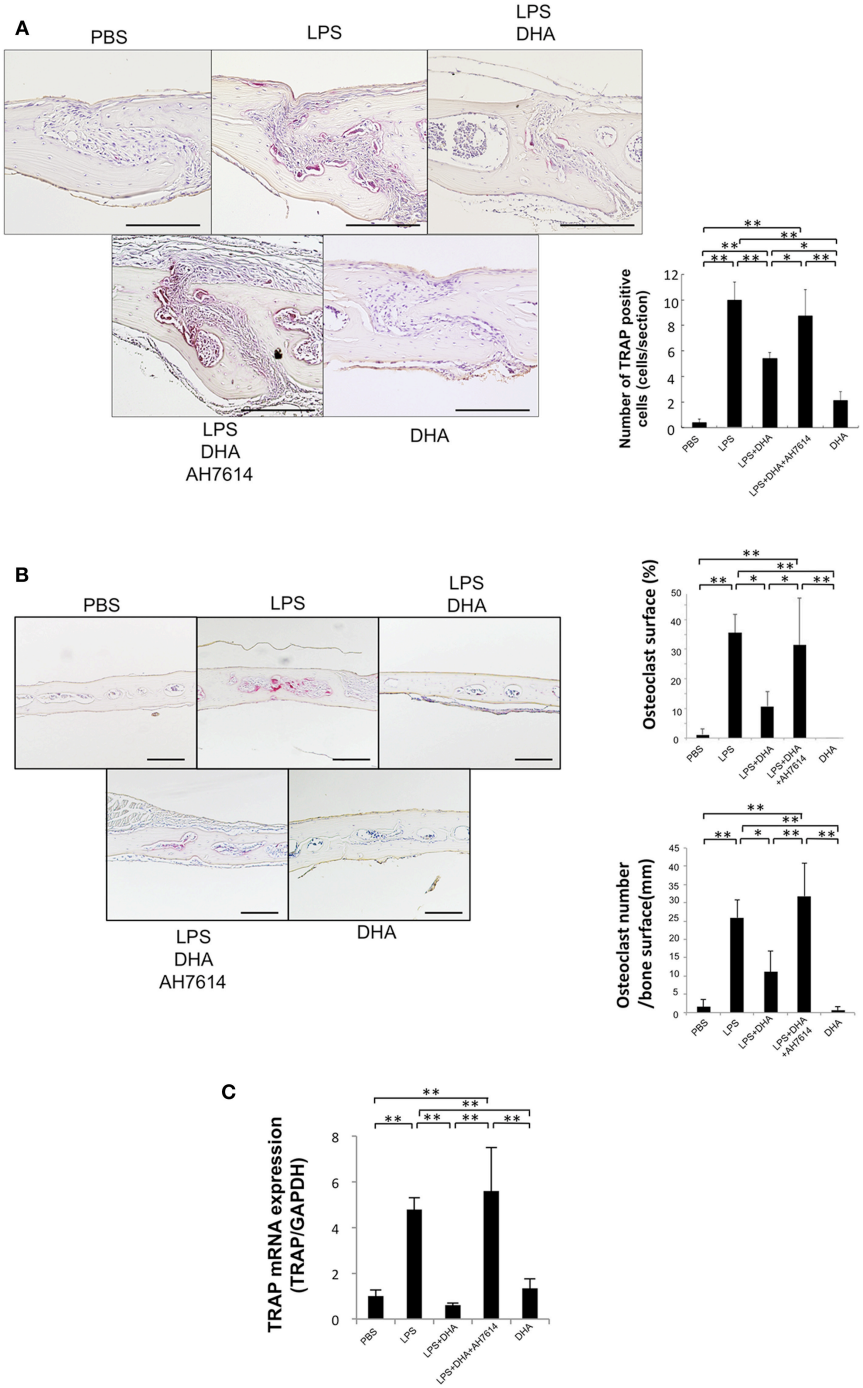
DHA inhibits LPS-induced osteoclast formation via GPR120 activation *in vivo*. **(A)** Histological sections of calvariae were prepared from C57/BL6 mice following 5 days of supracalvarial administration of PBS, LPS (100 μg/day), LPS (100 μg/day)+DHA (100 μg/day), LPS (100 μg/day)+DHA (100 μg/day)+AH7614 (100 μg/day), or DHA (100 μg/day). Sections were stained with TRAP solution and hematoxylin for counterstaining was performed. TRAP-positive cells appeared dark red. Scale bars = 100 μm. Numbers of TRAP-positive cells in the suture mesenchyme of calvariae from mouse groups administered PBS, LPS, LPS+DHA; LPS+DHA+AH7614 or DHA. Data are shown as means ± SD. Statistical significance was determined by Scheffe's test (*n* = 4; ^**^p < 0.01, ^*^p < 0.05). **(B)** Histological sections of calvariae after 5 days of daily supracalvarial administration of PBS, LPS (100 μg/day), LPS (100 μg/day)+DHA (100 μg/day), LPS (100 μg/day)+DHA (100 μg/day)+AH7614 (100 μg/day), or DHA (100 μg/day). Sections were stained with TRAP solution and hematoxylin for counterstaining was performed. The percentage of interface of bone marrow space covered by osteoclast and the number of TRAP-positive cells per millimeter of interface of bone marrow space were evaluated. Data is expressed as means ± standard deviation (SD). Statistical significance was determined by Scheffe's test (*n* = 4; ^**^p < 0.01 ^*^p < 0.05). **(C)** Expression levels of TRAP mRNA in calvariae of the mouse groups determined by real-time RT-PCR analysis. Data are expressed as means ± SD. Statistical significance was determined by one-way ANOVA and Turkey-Kramer tests (*n* = 4; ^**^p < 0.01, ^*^p < 0.05).

To assess whether LPS-induced osteoclast formation is inhibited by DHA via GPR120 *in vivo*, LPS and DHA with or without AH7614 were injected into mouse calvariae. When LPS was administered for 5 consecutive days, many large multinucleated TRAP-positive cells were noted within the suture mesenchyme of histological sections (Figure 2A), the higher percentage of interface of bone marrow space covered by osteoclasts and the higher number of osteoclast of interface of bone marrow space were observed (Figure 2B). In contrast, the mean number of TRAP-positive cells and the percentage of osteoclast area of interface of bone marrow space were markedly decreased in the LPS and DHA co-administered group compared with the LPS group, while the inhibitory effect of DHA was attenuated by co-administration with AH7614 (Figures 2A,B). Moreover, TRAP mRNA levels were remarkably lower in the LPS and DHA group than in LPS or LPS and DHA with AH7614 co-administered groups (Figure 2C).

### DHA Inhibits LPS-Induced Bone Resorption via GPR120 Activation *in vivo*

**Figure 3:**
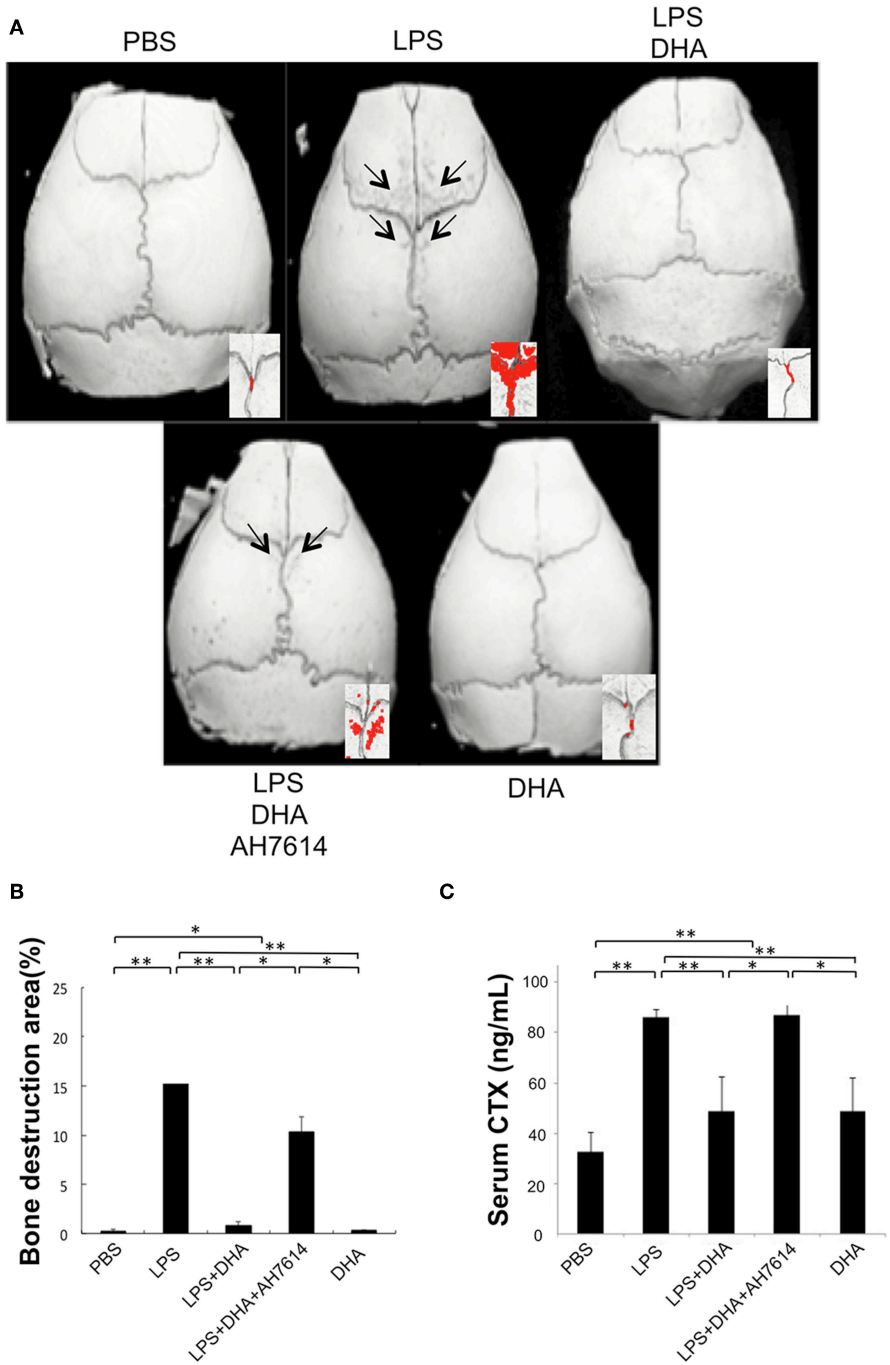
DHA inhibits LPS-induced bone resorption via GPR120 activation *in vivo*. **(A)** Micro-CT reconstruction images of calvariae. Images of calvariae resected from mice after 5 days of daily supracalvarial administration of PBS, LPS (100 μg/day), LPS (100 μg/day)+DHA (100 μg/day), LPS (100 μg/day)+DHA (100 μg/day)+AH7614 (100 μg/day), or DHA (100 μg/day). Arrows indicate bone resorption areas and red areas indicate extensive bone resorption. **(B)** Ratio of bone resorption area to total bone area. Results are expressed as means ± SD (*n* = 4; ^**^p < 0.01, ^*^p < 0.05). Differences were determined by Scheffe's test. **(C)** Serum CTX levels *in vivo*. Serum was prepared from mice following 5 days of daily supracalvarial administration of PBS, LPS (100 μg/day), LPS (100 μg/day)+DHA (100 μg/day), LPS (100 μg/day)+DHA (100 μg/day)+AH7614 (100 μg/day), or DHA (100 μg/day). Enzyme-linked immunosorbent assays was performed to determine concentrations of circulating CTX. Data are shown as means ± SD (*n* = 4; ^**^p < 0.01, ^*^p < 0.05). Differences were determined by one-way ANOVA and Turkey-Kramer tests.

The ratio of bone resorption area to total area in three-dimensional images of mouse calvariae was evaluated and compared in each group. Many bone destruction defects were observed in the LPS group compared with the PBS or DHA group. However, the bone destruction defects were significantly reduced in the LPS and DHA co-administered group, which was increased again in the LPS and DHA with AH7614 co-administered group (Figures 3A,B). We next evaluated serum CTX levels by using a mouse CTX assay kit. Serum CTX levels in the LPS group were significantly increased compared with those in the PBS or DHA group, although the LPS with DHA group had significantly lower CTX levels than the LPS group (Figure 3C). Additionally, serum CTX levels were significantly increased in the LPS and DHA with AH7614 co-administered group compared with the LPS and DHA group.

### DHA Inhibits Expression of LPS-Induced TNF-α and RANKL mRNAs *in vivo*

**Figure 4:**
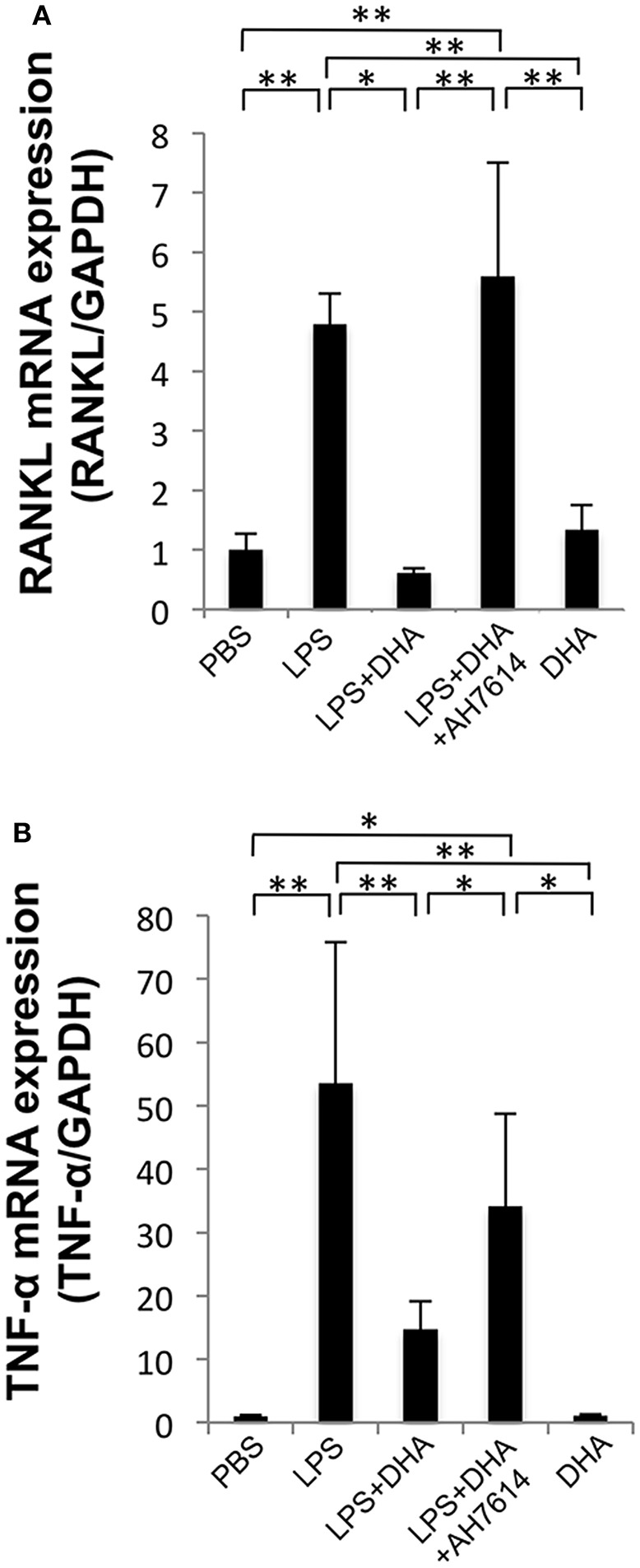
DHA inhibits expression of LPS-induced TNF-α and RANKL via GPR120 *in vivo*. **(A)** Expression levels of RANKL mRNA in mouse calvariae *in vivo*. **(B)** Expression levels of TNF-α mRNA in mouse calvariae *in vivo*. TNF-α and RANKL mRNA levels were determined using real-time RT-PCR. Total RNA from mouse calvariae was isolated after 5 days of daily supracalvarial injections of PBS, LPS (100 μg/day), LPS (100 μg/day)+DHA (100 μg/day) with or without AH7614 (100 μg/day) and DHA (100 μg/day). Expression levels of RANKL and TNF-α mRNA were normalized to that of GAPDH. Data are shown as means ± SD (*n* = 4; ^**^p < 0.01, ^*^p < 0.05). Differences were determined by one-way ANOVA and Tukey-Kramer tests.

The expression levels of osteoclast-related cytokines in calvariae were determined by using real-time RT-PCR (Figures 4A,B). RANKL and TNF-α mRNA levels were significantly elevated in the LPS group compared with the PBS group. In contrast, RANKL and TNF-α mRNA expression levels were significantly lower in the LPS and DHA group than those of the LPS group. Furthermore, treatment with GPR120 antagonist AH7614 elevated RANKL and TNF-α mRNA expression levels compared with the LPS and DHA group.

### DHA Suppresses LPS-Induced TNF-α Expression in Macrophages but Does Not Inhibit RANKL Expression in Osteoblasts

**Figure 5:**
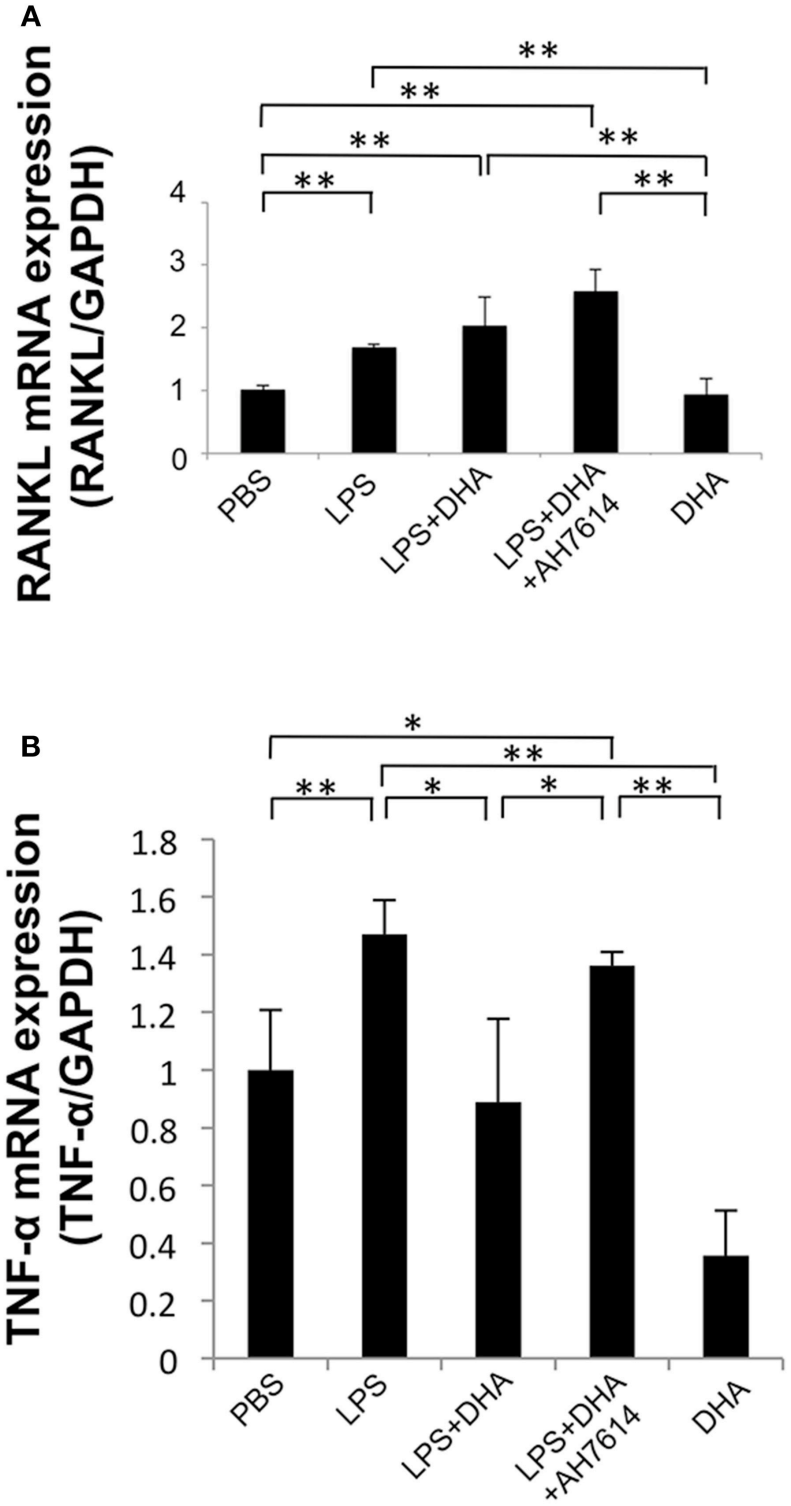
DHA suppresses LPS-induced TNF-α mRNA expression in macrophages but does not affect LPS-induced RANKL mRNA expression in osteoblasts. **(A)** Osteoblasts from cranial bone were incubated with PBS, LPS, LPS+DHA with or without AH7614, or DHA alone. After 24-h culture, total RNA was isolated and RANKL mRNA levels were determined using real-time RT-PCR. **(B)** Peritoneal macrophages were cultured with PBS, LPS, LPS+DHA with or without AH7614 or DHA alone. After 24-h culture, total RNA was isolated and TNF-α mRNA levels were determined using real-time RT-PCR. RANKL and TNF-α mRNA levels were normalized to that of GAPDH. Data are expressed as means ± SD (*n* = 4; ^**^p < 0.01, ^*^p < 0.05). Differences were determined by one-way ANOVA and Turkey-Kramer tests.

To precisely determine how DHA inhibits osteoclast formation via GPR120, we investigated RANKL mRNA expression levels in osteoblasts *in vitro*. RANKL mRNA expression levels were upregulated by LPS treatment compared with PBS or DHA alone. However, osteoblasts treated with both LPS and DHA demonstrated similar RANKL mRNA expression levels compared with cells treated with LPS alone. Moreover, RANKL mRNA expression levels were similar between the LPS and DHA with AH7614 co-administered group and the LPS and DHA group (Figure 5A).

We next investigated whether DHA inhibits LPS-induced TNF-α expression in macrophages *in vitro*. TNF-α mRNA expression levels were upregulated by LPS treatment, while they were decreased by LPS and DHA treatment. Moreover, treatment with GPR120 antagonist AH7614 significantly increased TNF-α mRNA expression levels compared with LPS and DHA (Figure 5B).

### DHA Does Not Suppress LPS-Induced Osteoclast Formation, Bone Resorption and Production of Osteoclast-Related Cytokines (TNF-α and RANKL) in *Ffar4*^(dE1/dE1)^ Mice

**Figure 6:**
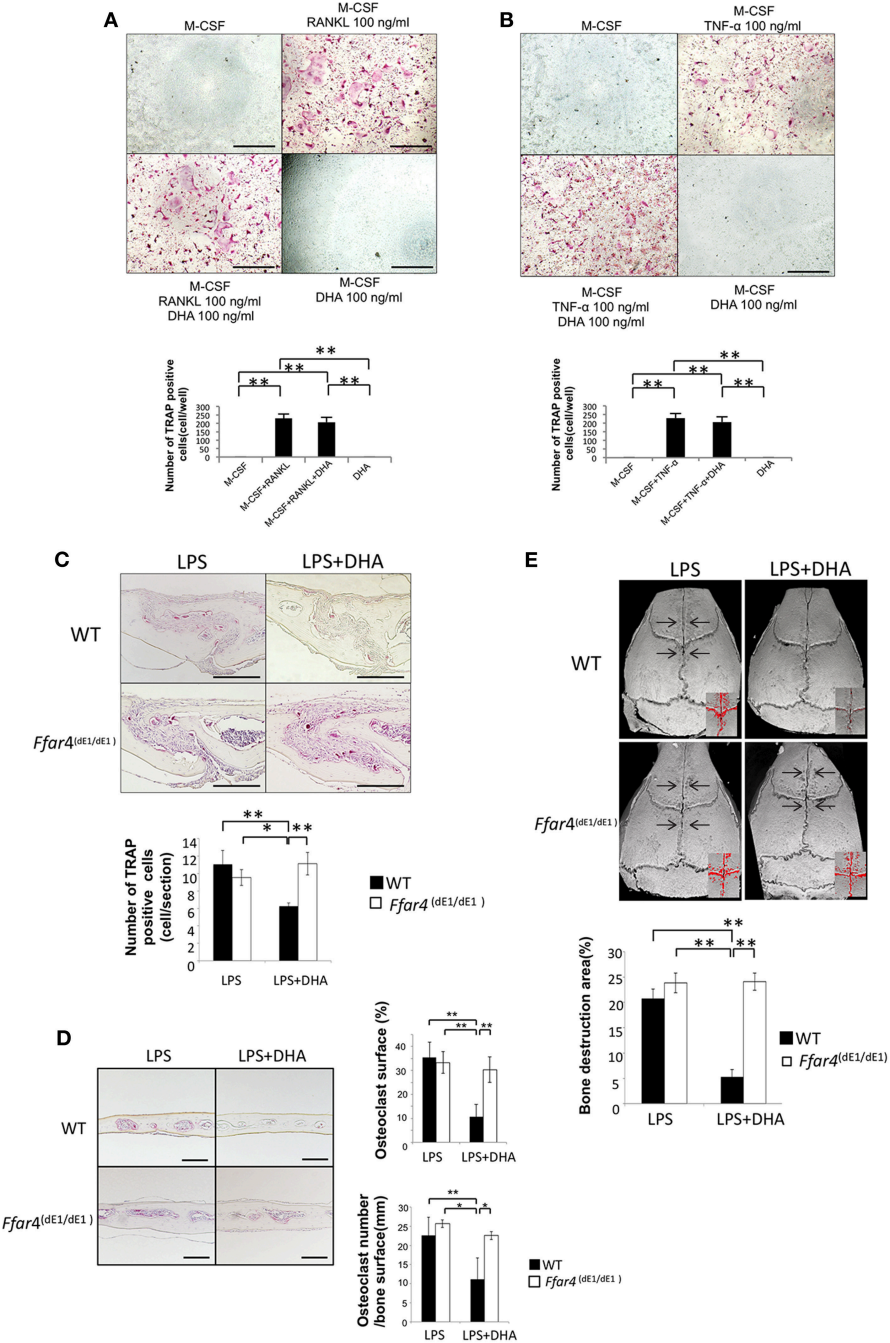
DHA does not suppress LPS-induced osteoclast formation, bone resorption and production of osteoclast-related cytokines (TNF-α and RANKL) in *Ffar4*^(dE1/dE1)^ mice. **(A)** Microscopic images and numbers of TRAP-positive cells. Osteoclast precursor cells from *Ffar4*^(dE1/dE1)^ mice were treated with M-CSF, M-CSF+RANKL with or without DHA or DHA alone. TRAP staining was performed and TRAP-positive cells were counted. **(B)** Microscopic images and numbers of TRAP-positive cells. Osteoclast precursor cells from *Ffar4*^(dE1/dE1)^ mice were treated with M-CSF, M-CSF+TNF-α with or without DHA or DHA alone, and then TRAP staining was performed. TRAP-positive cells were counted. Scale bars = 100 μm. **(C)** Histological sections of calvariae were prepared from *Ffar4*^(dE1/dE1)^ mice after 5 days of daily supracalvarial administration of LPS (100 μg/day) with or without DHA (100 μg/day). Sections were stained with TRAP solution and hematoxylin counterstaining was performed. TRAP-positive cells were counted. Scale bars = 100 μm. **(D)** Histological sections of calvariae were prepared from *Ffar4*^dE1/dE1^ mice after 5 days of daily supracalvarial administration of LPS (100 μg/day) with or without DHA (100 μg/day). The percentage of interface of bone marrow space covered by osteoclast and the number of TRAP-positive cell per millimeter of interface of bone marrow space were evaluated. **(E)** Micro-CT reconstruction images of calvariae. Images of calvariae excised from *Ffar4*^(dE1/dE1)^ mice after 5 days of daily supracalvarial administration of LPS (100 μg/day) with or without DHA (100 μg/day). Arrows indicate bone resorption areas and red areas indicate extensive bone resorption. Data are shown as means ± SD (*n* = 4; ^**^p < 0.01, ^*^p < 0.05). Differences were determined by Scheffe's test.

To clarify the role of GPR120 in osteoclast formation, we generated and used *Ffar4* knockout mice [*Ffar4*^(dE1/dE1)^]. Osteoclast precursor cells from *Ffar4*^(dE1/dE1)^ mice were treated with M-CSF, M-CSF, and RANKL with or without DHA or DHA alone, and then the number of TRAP-positive cells was counted. The number of TRAP-positive cells was increased in the M-CSF and RANKL group. However, there was no significant difference in the number of TRAP-positive cells between the M-CSF and RANKL with DHA group and the RANKL group (Figure 6A). Similar results were observed in osteoclast formation following TNF-α and DHA treatment (Figure 6B).

We also evaluated osteoclast formation *in vivo* using *Ffar4*^(dE1/dE1)^ mice. Supracalvarial administration of LPS with or without DHA was carried out for 5 days, and then the number of TRAP-positive cells at the mesenchyme of the sagittal suture was counted. Furthermore, the percentage of interface of bone marrow space covered by osteoclasts and the number of osteoclast of interface of bone marrow space were evaluated. In the LPS group, a great number of TRAP-positive cells and the higher percentage of osteoclast area of interface of bone marrow space was observed in both wild type and *Ffar4*^(dE1/dE1)^ mice. There was no difference in the number of TRAP-positive cells and the percentage of osteoclast area of interface of bone marrow space between the LPS and DHA co-administrated group and the LPS group. However, the number of TRAP-positive cells and percentage of osteoclast area of interface of bone marrow space were decreased in the LPS and DHA co-administrated group compared with that of wild type mice (Figures 6C,D). There is no significant difference of osteoclast number and percentage of osteoclast area in both PBS injected wild type and *Ffar4*^(dE1/dE1)^ mice as control (Supplementary Figure 2).

Finally, we investigated bone resorption in *Ffar4*^(dE1/dE1)^ mice by using microfocus CT. There was no significant difference between the ratio of bone resorption area in wild type and *Ffar4*^(dE1/dE1)^ mice administered LPS alone. In contrast, a marked decrease was observed in the ratio of bone resorption area in wild type mice co-administered LPS and DHA, while no significant change was observed in *Ffar4*^(dE1/dE1)^ mice co-administered LPS and DHA compared with respective mice that received LPS alone (Figure 6E).

## Discussion

Favorable effect of food intake and supplement of n-3 fatty acids such as DHA were provided against skeletal disorders such as osteoporosis and rheumatoid arthritis (19, 20). There are some *in vitro* studies of effect of DHA on osteoclast formation by using RAW264.7 cell (34, 39) and human CD14+ monocytes (33). GPR120, which is one of the cell surface receptor, has an important role in the regulation of inflammation. Therefore, GPR120 is being watched with keep interest as one of the potential therapeutic target in several inflammatory disorders. In this study, we showed that DHA inhibits LPS-induced osteoclast formation and bone resorption *in vivo* via GPR120 by inhibiting LPS-induced TNF-α production in macrophages and directly inhibiting osteoclast formation.

In the present study, we focused on GPR120, because several studies showed GPR120 is highly expressed in RAW264.7 cells, intraperitoneal macrophages, osteoclast precursors and osteoclast (27, 40, 41). It has been reported that DHA can activate not only GPR120 but also GPR40 (42). GPR40 activation by using its selective agonist GW9508 by blocked osteoclast formation (43). However, Kim et al. reported that GPR120 was highly expressed but GPR40 is very low in osteoclast precursors and osteoclast (41). Therefore, we thought that GPR120 might play important role in osteoclast formation and function.

Several studies showed that DHA inhibits osteoclast formation *in vitro*. Kim et al. reported that DHA inhibits RANKL-induced osteoclast formation in primary murine macrophages by suppressing the activation of NF-kB and MAPKs (44). Sun et al. also showed that DHA inhibits RANKL-induced osteoclast formation in bone marrow macrophages (32). Furthermore, DHA reduces RANKL-induced osteoclast formation in RAW 264.7 cells (34, 39) by inhibition of c-Fos expression (34). It has also been reported that DHA inhibits osteoclast formation and function in human CD14+ monocytes (33). In the present study, we initially investigated whether DHA can inhibit RANKL- and TNF-α-induced osteoclast formation by directly affecting osteoclast precursors *in vitro*. Our results indicated that DHA directly inhibited RANKL- and TNF-α-induced differentiation of osteoclast precursors. In addition, we investigated whether DHA inhibits RANKL- and TNF-α-induced osteoclast formation via GPR120 activation by using GPR120 antagonist AH7614 *in vitro*. AH7614 dose-dependently increased the number of TRAP-positive cells in both RANKL- and TNF-α-induced osteoclast formation in cells cultured with DHA. These results suggested that DHA inhibits osteoclast formation via GPR120 *in vitro*.

It has been reported that DHA inhibits bone loss in ovariectomized mice owing to its inhibition of osteoclast generation and activation *in vivo* (32). In addition, perinatal DHA supplementation is associated with decreased bone resorption, decreased osteoclast density and a higher bone mass in rats (45). Periodontitis rats, which are generated by oral infection of *Porphyromonas gingivalis*, treated with fish oil containing DHA had signified less alveolar bone resorption (46). In this study, DHA inhibited osteoclast formation induced by LPS. Daily supracalvarial administration of 100 μg of DHA for 5 days inhibited LPS-induced osteoclast formation *in vivo*. The inhibitory effect of DHA on bone resorption induced by LPS was also evaluated. The increase in bone resorption was evaluated using the ratio of the bone resorption area to total area in microfocus CT images. We also determined bone destruction by evaluating the serum CTX level. The increase in bone resorption was significantly lower in the LPS and DHA group than in the LPS group. These results indicated that DHA inhibits LPS-induced osteoclast formation and bone destruction *in vivo*. The results of the effect of DHA on osteoclast formation are similar to those reported in previous studies (33, 34, 39, 44). Furthermore, AH7614 increased osteoclast formation and bone resorption in the LPS and DHA group. Taken together, these results indicated that DHA inhibits LPS-induced osteoclast formation via GPR120 *in vivo*.

Several reports showed that DHA inhibits pro-inflammatory cytokine production from several cell types. DHA can inhibit TNF-α and interleukin (IL)-6 production in primary mouse macrophages and RAW 247.6 cells by binding to GPR120 (27). DHA also reduces LPS-induced production of pro-inflammatory cytokines, such as TNF-α, IL-1β, and IL-6, in THP-1 macrophages (47). Furthermore, DHA reduces IL-1β expression in bone marrow-derived macrophages (48). We suspect that the mechanisms of inhibitory effect of LPS-induced osteoclast formation and bone resorption *in vivo* by DHA are conceivable via either of two possible mechanisms. One possible mechanism is DHA blocks expression of LPS-induced cytokines associated with osteoclast formation. It is well-known that RANKL and TNF-α are important factors in osteoclast formation (2, 6, 7). Several groups showed that LPS is able to induce TNF-α and RANKL expression *in vivo* (13, 49, 50). In the present study, TNF-α and RANKL mRNA expression levels were increased in LPS-treated mice. On the other hand, TNF-α and RANKL mRNA expression was suppressed in DHA and LPS co-administered mice in comparison with LPS-treated mice. Our results were consistent with the hypothesis that DHA inhibits osteoclast formation by suppressing the expression of LPS-induced production of osteoclast-associated cytokines. Another possible mechanism of DHA inhibition is that DHA directly suppresses osteoclast formation by influencing cell differentiation. Our results indicated that DHA directly inhibited both RANKL- and TNF-α-induced differentiation of osteoclast precursor cells into osteoclasts. Moreover, AH7614 increased the concentration of osteoclast-related cytokines in LPS and DHA-treated mice. The results suggested that DHA inhibited LPS-induced production of osteoclast-related cytokines via GPR120 *in vivo*. These results indicated that the inhibitory effects of DHA on osteoclast formation by LPS *in vivo* are due to both decreased production of osteoclast-associated cytokines and direct actions of DHA on osteoclast precursors through GPR120.

Next, we evaluated whether DHA inhibits LPS-induced TNF-α mRNA expression in macrophages. The results indicated that downregulation of TNF-α mRNA by DHA might result from a direct action of DHA on macrophages. AH7614 increased LPS-induced TNF-α mRNA expression in macrophages with DHA treatment. Therefore, the results suggested that DHA inhibited LPS-induced TNF-α in macrophages via GPR120. In addition, we investigated the influence of DHA on LPS-induced RANKL expression in osteoblasts, but no effect of DHA on LPS-induced RANKL expression was found. Our results suggested that DHA likely suppresses LPS-induced osteoclast formation *in vivo* by directly inhibiting LPS-induced TNF-α expression in macrophages. This inhibition subsequently blocks RANKL expression in osteoblasts, although there is no effect of DHA on LPS-induced RANKL expression in osteoblasts, in addition to direct inhibition of osteoclast formation.

Finally, we evaluated whether both RANKL- and TNF-α-induced osteoclast formation is inhibited by DHA through GPR120 and whether DHA inhibits LPS-induced osteoclast formation and bone resorption *in vivo* via GPR120 by using GPR120-deficient mice. The effects of DHA on osteoclast formation *in vitro* disappeared by using osteoclast precursors from GPR120-deficient mice and inhibition of LPS-induced osteoclast formation and bone resorption by DHA *in vivo* disappeared in GPR120-deficient mice. The results strongly suggested that the effect of DHA on osteoclast formation *in vivo* and *in vitro* was maintained via GPR120.

**Figure 7:**
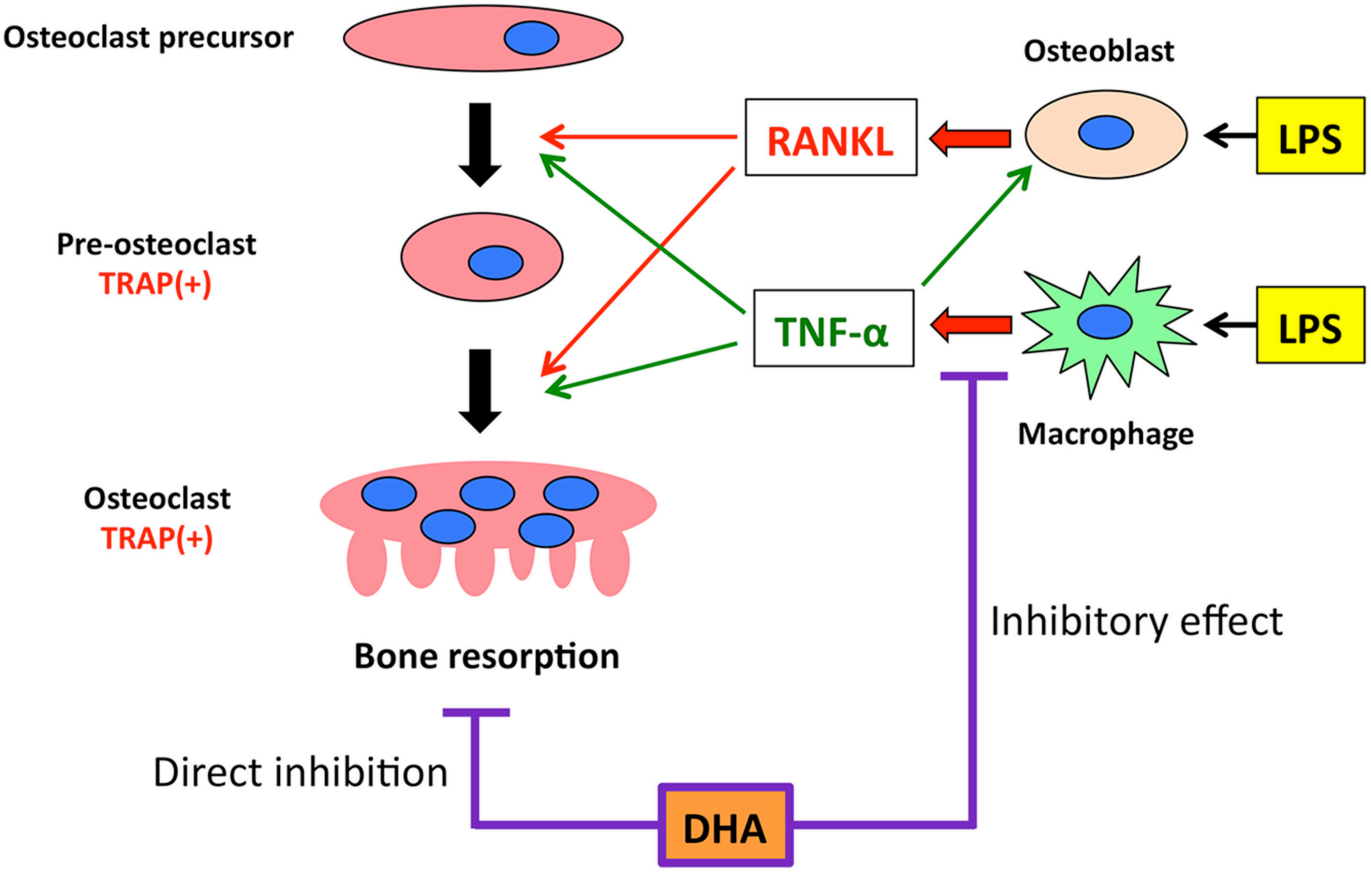
Putative model of the inhibitory effect of GPR120 activation by DHA in LPS-induced osteoclast formation and production of osteoclast-related cytokines (RANKL and TNF-α) *in vivo*. Both RANKL and TNF-α have essential roles in inducing osteoclast differentiation. When osteoclast precursors are treated with RANKL, TRAP-positive pre-osteoclasts are induced. These cells fuse and differentiate into osteoclasts under the influence of RANKL, subsequently inducing bone resorption. TNF-α acts in a similar manner as RANKL to induce osteoclast differentiation. These cytokines also affect LPS-induced osteoclast formation and bone resorption *in vivo*. LPS administration induces RANKL expression in osteoblasts and TNF-α expression in macrophages. Furthermore, LPS-induced TNF-α production increases RANKL expression in osteoblasts. GPR120 activation directly inhibits osteoclast precursor differentiation and LPS-induced TNF-α expression in macrophages. However, the inhibitory effect of GPR120 may be unrelated to RANKL expression in osteoblasts.

In conclusion, we demonstrated that DHA inhibits LPS-induced osteoclast formation and bone destruction *in vivo* and directly inhibits RANKL- and TNF-α-induced osteoclast formation *in vitro*. The underlying mechanisms by which DHA inhibits LPS-induced osteoclast formation and bone destruction *in vivo* were associated with its inhibition of LPS-induced TNF-α production in macrophages and direct inhibition of RANKL- and TNF-α-induced osteoclast formation via GPR120 (Figure 7).

## Author Contributions

AK and HK contributed to conception, design, data acquisition, data analysis, data interpretation, and drafting of the manuscript. HK and AI contributed to critical revision of the manuscript. KK, SO, JQ, W-RS, FO, TN, AM, and YN collected the samples and performed data analyses. HK and IM supervised the project. All authors provided final approval and agree to be accountable for all aspects of the work.

### Conflict of Interest Statement

The authors declare that the research was conducted in the absence of any commercial or financial relationships that could be construed as a potential conflict of interest.
