# Supplementary material for: Docosahexaenoic Acid Inhibits Inflammation-Induced Osteoclast Formation and Bone Resorption in vivo Through GPR120 by Inhibiting TNF-α Production in Macrophages and Directly Inhibiting Osteoclast Formation
Source: Front Endocrinol (Lausanne). 2019 Mar 15;10:157. doi: 10.3389/fendo.2019.00157 (PMC6436080; doi:10.3389/fendo.2019.00157)
Supplement: Supplementary file 1 [file Data_Sheet_1.PDF]

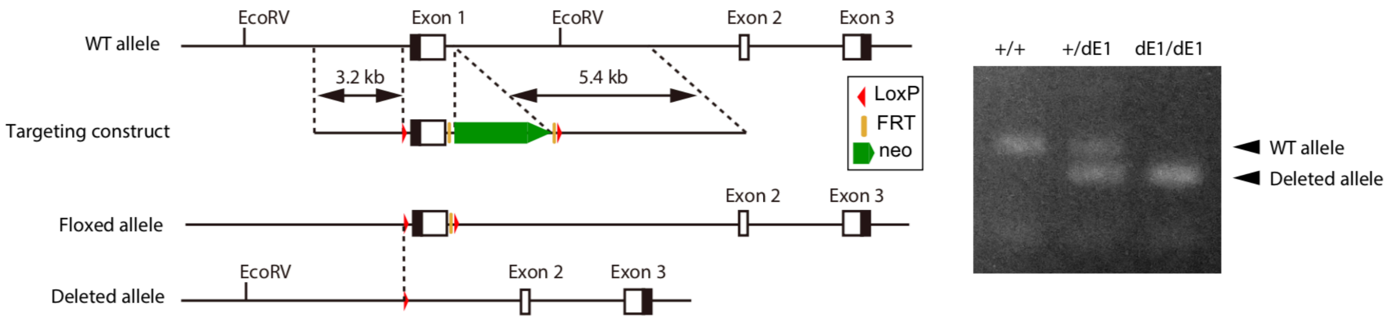

Supplementary Figure 1. Schematic illustration of *Ffar*4-targeting vector, *Ffar*4-floxed allele and recombined allele (Ffar4dE1). Red arrows indicate loxP site. *Ffar*4-floxed mice were crossed with transgenic mice expressing Cre-recombinase under the control of the CAG promoter. All mice were genotyped by PCR requires use of three primers as follows; primer 1: CAC TCT TCG ATC AGG GTT AG, primer 2: GAA ACT CTG TGA GAA GCC ACC TCG, primer 3:ATC TGC GAG GTA ACT GAA TG.

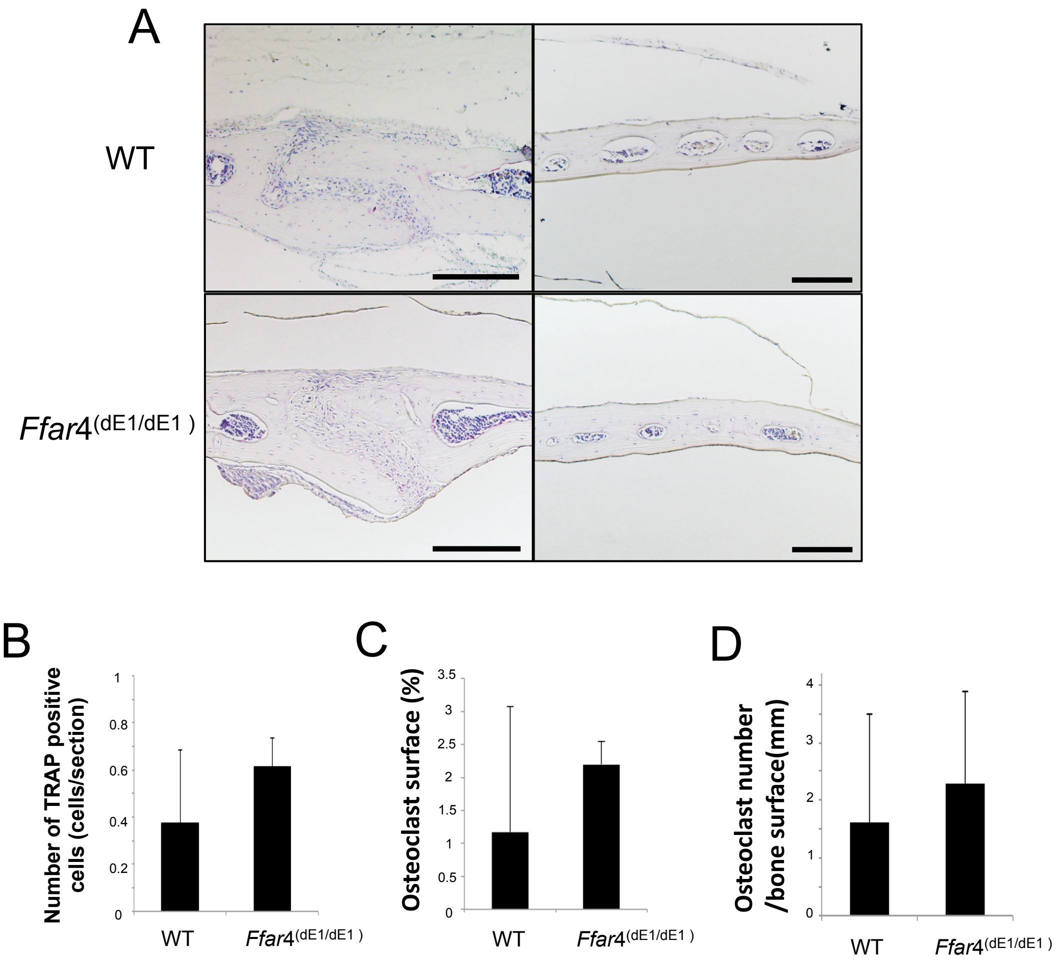

Supplementary Figure 2. (A) Histological sections of calvariae obtained from wild type and *Ffar4*$^{(dE1/dE1)}$ mice after five days of daily supracalvarial administration with PBS. Scale bars =100 µm. (B) The numbers of TRAP-positive cells in the suture mesenchyme of calvariae obtained from wild type and *Ffar4*$^{(dE1/dE1)}$ mice. (C) The percentage of interface of bone marrow space covered by osteoclast. (D) The number of TRAP-positive cell per millimetre of interface of bone marrow space. (B)-(D) Results are expressed as means±SD. Differences were determined using Scheffe's test.
